# The Association Between Physical Activity and Mathematical Achievement Among Chinese Fourth Graders: A Moderated Moderated-Mediation Model

**DOI:** 10.3389/fpsyg.2022.862666

**Published:** 2022-05-09

**Authors:** Jing Zhou, Hongyun Liu, Hongbo Wen, Xiuna Wang, Yehui Wang, Tao Yang

**Affiliations:** ^1^Collaborative Innovation Center of Assessment Toward Basic Education Quality, Beijing Normal University, Beijing, China; ^2^Faculty of Psychology, Beijing Normal University, Beijing, China; ^3^Beijing Key Laboratory of Applied Experimental Psychology, Faculty of Psychology, National Demonstration Center for Experimental Psychology Education, Beijing Normal University, Beijing, China

**Keywords:** mathematical achievement, mathematical anxiety, physical activity, parental support, conditional process model, moderated moderated-mediation model

## Abstract

This study explored the association between out-of-school physical activity (PA) and mathematical achievement in relation to mathematical anxiety (MA), as well as the influence of parents’ support for their children’s physical activity on this association, to examine whether parental support for physical activity affects mental health and academic performance. Data were collected from the responses of 22,509 (52.9% boys) children in Grade 4 from six provinces across eastern, central, and western China who completed the mathematics component and the physical education and health component of the national-level education quality assessment. A moderated moderated-mediation model was tested using PROCESS v3.4 and SPSS v19.0, with socioeconomic status, school location, and body mass index as controlled variables. Out-of-school physical activity had a positive effect on children’s mathematical achievement, and math anxiety partially mediated this association. The indices of conditional moderated mediation through the parental support of both girls and boys were, respectively, significant, indicating that children can benefit from physical activity, and that increased perceived parental support for physical activity can alleviate their children’s math anxiety and improve their mathematics, regardless of gender. However, gender differences were observed in the influence of parental support for physical activity on anxiety: Although girls’ math anxiety levels were significantly higher, the anxiety levels of girls with high parental support were significantly lower than those of boys with low parental support.

## Introduction

Physical activity (PA) refers to any bodily movement produced by skeletal muscles that requires energy expenditure (Donnelly et al., 2016) and plays a vital role in the growth and health of children; however, it has not received the attention it deserves. The World Health Organization’s survey of 72,845 schoolchildren from 34 countries showed that most children did not get enough exercise, while nearly a third were sedentary (Guthold et al., 2010). Moreover, studies related to physical activity have also indicated that many children do not meet national physical activity guidelines—from a systematic review and meta-analysis (Watson et al., 2017). Although schools provide the ideal environment for promoting children’s physical activity, it is difficult to increase physical activity during school days due to factors such as competition among the key learning areas and the limited time children spend in school. The above survey also revealed that parents do not pay sufficient attention to physical activity after school. In countries or regions with high academic pressure in particular, though parents may know that physical activity is a key factor for their children’s growth and health, they may still only prioritize it after academic performance when the time available is limited. Thus, it can be seen that it is challenging to integrate physical activity into children’s daily lives.

In fact, empirical studies have reported that physical activity aids not only children’s physical and mental health but also their academic achievement (Ashcraft, 2002; Wang et al., 2019; Hermassi et al., 2021). While related research has covered several academic areas (e.g., language, mathematics, spelling, reading, science, and geography), mathematics has been studied the most and the results are diverse (Sibley and Etnier, 2003; Esteban-Cornejo et al., 2015; de Greeff et al., 2018; Bedard et al., 2019; Barbosa et al., 2020). In general, physical activity is an effective way of enhancing physical and mental health. Research has shown that the potential benefits of physical activity on cognitive performance, learning, brain structure and brain function may be the foundation for improved academic performance (Donnelly et al., 2016). Evidence suggests that physical activity improves physiology, like executive function (EF), which in turn improves academic performance (Álvarez-Bueno et al., 2017; de Greeff et al., 2018; Ishihara et al., 2018b). However, the relationship between physical activity and mental health is not a simple causal link and can be affected by other factors (Lagerberg, 2005).

In addition, the research on the impact of physical activity on academic performance has been inconsistent. On the one hand, the extant research has considered physical activity from different perspectives, such as enhanced and enriched physical activity, or aerobic, acute, and chronic exercise (Petruzzello et al., 1991; Álvarez-Bueno et al., 2017). On the other hand, most of the research on this association has focused on physical activity in schools, such as curricular physical education (PE), extracurricular physical activity (active recess, or lunch time or sports programs), and integrated physical activity (active breaks or teaching subjects such as math through physical activities), and little attention has been given to out-of-school physical activity. This diversity of research perspectives makes it extremely valuable to further explore the impact of physical activity on children’s mental health and academic performance.

Many typical psychological factors have been used as intermediary variables to explore the influence mechanism of physical activity on mental health and academic performance. The results are diverse and should be further verified and enriched (Tremblay et al., 2000; Dapp and Roebers, 2019). The distraction hypothesis provides a basis for exploration, maintaining that diversion from unpleasant stimuli or painful somatic symptoms leads to effect improvements following exercise sessions (Greist et al., 1979; Morgan, 1985; Hill, 1987; Paluska and Schwenk, 2000). Research results have demonstrated that many psychological factors play a mediating role between physical activity and academic performance (Tremblay et al., 2000; Sigfusdottir et al., 2007; Dapp and Roebers, 2019; Wang et al., 2019); however, it remains necessary to add more psychological intermediary factors to the analysis to provide more reliable evidence in support of the distraction hypothesis.

Furthermore, according to social cognitive theory, strong social support networks increase an individual’s self-efficacy, thereby allowing them to overcome barriers to being physically active (Bandura, 1999). As an indispensable social support in childhood, parents are likely to provide the support necessary for participation in physical activity when they value the outcomes associated with regular physical activity. This idea is consistent with the central tenets of the major attitude–behavior theories (theory of reasoned action, planned behavior, and social cognitive theory) (Trost et al., 2003). Studies have explored the role of parental support for physical activity among boys and girls (Brustad, 1996; Henriksen et al., 2016; Lijuan et al., 2017), thus far, little research has comprehensively examined the relationship between physical activity and academic achievement in relation to mental health, or the influence of parental support for their children’s physical activity on this association. Since this relationship remains unclear, the current research explores this issue.

What kind of influence does the parental support for physical activity perceived by a boy or girl have on their mental health and academic performance? If parental support for physical activity is confirmed to promote mental health and academic performance, this will enable parents and education departments to not only further realize the potential value of physical activity for improved mental health and academic performance but also increase the level of support for physical activity in terms of opinions, awareness, and behaviors and strengthen the communication between school and families. This support for physical activity will increase the expected positive effects, which will prove valuable for children’s growth, mental health, and academic performance.

## Literature Review

### Out-of-School Physical Activity and Mathematics

The effectiveness of physical activity interventions for promoting children and adolescents’ cognition and academic achievement has been reported since 1997 (Etnier et al., 1997; Donnelly et al., 2009, 2016; Langford et al., 2014; Diamond, 2015; Esteban-Cornejo et al., 2015). In recent years, more than 200 studies have explored the association between physical activity and academic success in school-aged children. It can, thus, be concluded that there is a significant positive relationship between physical activity and cognitive functioning in children (Esteban-Cornejo et al., 2015; Donnelly et al., 2016). A number of exercise and brain experiments have clearly shown that regular physical activity alters specific brain structures and functions, particularly in tests that require more executive function (EF) (Miyake et al., 2000; Diamond, 2015; Gunnell et al., 2019). This provides evidence for the executive function hypothesis, which says that exercise has the potential to induce vascularization, nerve growth, and altered synaptic transmission, thereby modifying thinking, decision-making, and behavior in brain regions associated with EF (Kopp, 2012). In addition, physical activity can also help mental health by improving skeletal and musculoskeletal functions to help relieve or reduce depression, anxiety, and stress (Eveland-Sayers et al., 2009; Teferi, 2020). The mechanism of the association between physical activity, cognition, and mental health can be explained by a conceptual model hypothesis, that is, a neurobiological, psychosocial, and/or behavioral linkage mechanism and may be affected by the frequency, intensity, timing, type and context of physical activity (Lubans et al., 2016; Barth Vedøy et al., 2020).

So far, systematic reviews show no indication that increases in physical activity negatively affect cognition or academic achievement (Donnelly et al., 2016). The overall effect of different modes of physical activity had null or small to medium effects on academic achievement (Barbosa et al., 2020). The findings varied due to the diversity of cognitive domains and physical activity indicators involved (Esteban-Cornejo et al., 2015; Donnelly et al., 2016; de Greeff et al., 2018; Barbosa et al., 2020; Wassenaar et al., 2020). And inconsistent results were observed across multiple subject areas, including mathematics, reading, language (Fedewa and Ahn, 2011; Martin et al., 2014), science, spelling, and geography (de Greeff et al., 2018; Bedard et al., 2019).

Compared with other disciplines, the discussion on the relationship between physical activity and mathematics attracted more attention (Barbosa et al., 2020). Indeed, mathematics has an irreplaceable position as a fundamental and important discipline. In China, mathematics has become a compulsory subject in grades 1-12, and is a compulsory subject for taking college entrance examinations and senior high school entrance examinations. It strongly supports other science and engineering disciplines and subsequent learning practices. This explains why researchers pay more attention to mathematics when exploring the relationship between physical activity and academic performance (Fedewa and Ahn, 2011). Although most studies have documented that physical activity has a positive effect on mathematics (Ericsson and Karlsson, 2014; Mullender-Wijnsma et al., 2015; Riley et al., 2016), some results are contradictory (Sallis et al., 1999; Reed et al., 2010; Tarp et al., 2016), with null or small to medium effects (Barbosa et al., 2020).

In addition, most of the relevant research focused on intramural sports, with little attention paid to extramural sports. Systematic review studies have shown that most studies on physical activity have focused on physical education and active tasks in schools, revealing that math-related skills benefit from both curricular and integrated PE, as well as from extracurricular physical activity. Few studies focused on out-of-school physical activity, and there is a lack of research on the impact of out-of-school physical activity on academic performance (Álvarez-Bueno et al., 2017). The different perspectives taken to explore physical activity are one of the key reasons for the inconsistent results in the literature. Moreover, most of the previous research samples have not been large samples, posing limitations in sample representation and to our understanding of the large-scale situation. Does a large sample of data provide evidence of the positive effect of out-of-school physical activity on mathematics? This study attempts to fill these gaps.

### Mediating Effect of Math Anxiety Between Physical Activity and Mathematics

In order to further explore the inconsistent relationship between physical activity and mathematics, researchers have delved into the influencing factors according to the above-mentioned influence mechanism. For example, the mediating physiological, cognitive and motor variables (e.g., aerobic and physical fitness; Ashcraft, 2002; Wang et al., 2019), and psychological variables also are important aspects that researchers focus on (Tremblay et al., 2000; Dapp and Roebers, 2019; Kyan et al., 2019). Based on the psychological mechanisms of the distraction hypothesis (Greist et al., 1979; Morgan, 1985; Hill, 1987; Paluska and Schwenk, 2000), some classical psychological variables have been analyzed, such as self-concept, self-confidence and interest, self-efficacy, self-esteem, motivation, and depressive mood. The mediating roles of different psychological factors between physical activity and academic performance have been confirmed over time—one variable after another (Tremblay et al., 2000; Sigfusdottir et al., 2007; Dapp and Roebers, 2019; Kyan et al., 2019; Wang et al., 2019). However, as one of the most common mental health problems (Kessler et al., 2012), anxiety has not been included among these psychological variables.

The ability of exercise to relieve anxiety has been validated by some meta-analyses (Long and van Stavel, 1995; Guszkowska, 2004). Within the framework of self-decision theory, researchers have observed that behaviors that promote the satisfaction of individuals’ basic psychological needs can reduce social anxiety to a certain degree (Brunet and Sabiston, 2009), with physical activity representing one such behavior (Ren and Li, 2020). In similar results, frequent physical activity was associated with lower levels of depression and anxiety and a greater sense of well-being (McMahon et al., 2017). Nevertheless, since the concept of physical activity differs across the literature and the potential influencing factors have not been considered, the association between physical activity and anxiety remains unclear (Williams et al., 2016; Kandola et al., 2018).

Meanwhile, mathematics anxiety (MA), defined as fear and worry related to math stimuli and situations (Richardson and Suinn, 1972), is related to poor performance on mathematics achievement tests (Hembree, 1990). Meta-analyses from the 1990s previously established a significant, small-to-moderate, and negative correlation between math achievement and math anxiety (Ma, 1999; Barroso et al., 2021). Although anxiety has been employed as an intermediary variable that has a negative effect on mathematics (Liu et al., 2020), few studies have applied anxiety as a mediator to assess the effects of physical activity on academic achievement. Does math anxiety play a mediating role between out-of-school physical activity and mathematics? This is the second question this study attempts to explore.

### Moderating Role of Parental Support According to Gender

There is a non-negligible relationship between out-of-school physical activity and family factors for primary school students. And as one of the most important social supports in childhood, parental support plays a necessary role in children’s physical activity behaviors (Trost et al., 2003; Van Der Horst et al., 2007; Hennessy et al., 2010; Bassett-Gunter et al., 2017). However, the results regarding the association between parental support and children’s physical activity levels have been inconclusive (Gustafson and Rhodes, 2006). This inconsistency can be attributed to the different measures of social support used, their differing reliability and validity, and the different modes of physical activity measurement adopted (Prochaska et al., 2002).

The positive effect of parental support on alleviating anxiety has previously been identified (Ma, 1999; Ren and Li, 2020). There is also evidence that social support moderates psychological distress (Solberg and Viliarreal, 1997; Constantine et al., 2003) and that the dimensions of perceived social support (including familial support) exert a mediating effect on social anxiety in sports and play a moderating role in the relationship between physical exercise and social anxiety (Ren and Li, 2020), although the moderating role of social support in previous studies was not specifically related to physical activity. Does parental support for physical activity perceived by children play a moderating role in the relationship between physical activity and mathematics through math anxiety? This study believes that this is another question worthy of further exploration.

Moreover, gender also constitutes an influencing factor in relation to physical activity (Kremers et al., 2007; Simen-Kapeu and Veugelers, 2010; Kyan et al., 2019), exerting a direct or indirect relationship between parental social support and children’s physical activity in a manner that differs between genders (Peterson et al., 2013). However, in one report on the critical nature of activity-related support from family and friends, no gender differences in the impact of activity-related support were identified (Davison, 2004). Another review reported that there were significant correlations between parental support and children’s physical activity levels, although the results regarding an association between parental and child physical activity levels were mixed (Gustafson and Rhodes, 2006). Thus, the role of parental support in different gender groups deserves further attention.

The results on gender difference (between male, female, and non-binary individuals; Ahn and Fedewa, 2011) as a moderator in relation to physical activity and mental health have been significant (Ishihara et al., 2018a). However, in a previous study on gender, anxiety, and social support, the three-way interaction between gender and anxiety was such that boys and girls differed with respect to perceived social support but not anxiety (Landman-Peeters et al., 2005). In general, the findings regarding gender as a moderator in the relationship between physical activity and mental health have been inconsistent (Ishihara et al., 2018a). Does gender have a secondary moderating effect on the moderated-mediation model? For a deeper exploration of the crucial role of the parent–child relationship in children’s math anxiety (Ren and Li, 2020; Ma et al., 2021), gender differences in the benefits of physical activity for their mental health warrant further study.

In addition, socio-economic status (SES) and school location are also important factors influencing academic performance (Alordiah et al., 2015). Body mass index (BMI; calculated using a formula to determine an individual’s height and body weight index) is a key indicator used in the physical activity-related research (Hansen et al., 2014; Torrijos-Niño et al., 2014; Li Y. et al., 2020). These variables would be effectively controlled in the moderated moderated-mediation relationship explored in the present study.

### The Present Study

**FIGURE 1:**
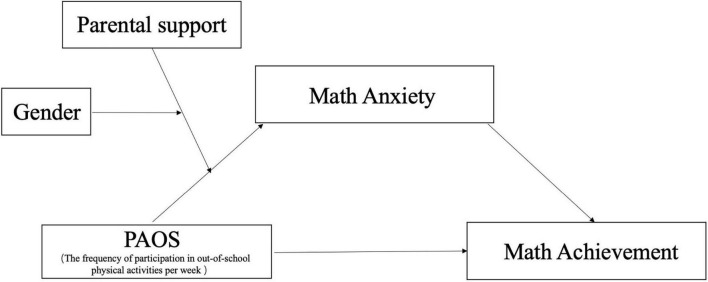
Proposed theoretical framework.

In summary, few studies in the past have explored (a) the mediating effect of math anxiety between out-of-school physical activity and mathematics, or (b) the double moderating effect of children’s perceived parental support for their physical activity in terms of different genders on this mediation effect. The results of such an analysis can act as a valuable reference for the practical promotion of children’s physical activity by schools and families. A moderated moderated-mediation model based on these four research questions was investigated (Figure 1), with perceived parental support for physical activity acting as the primary moderator *W* and gender acting as the secondary moderator *Z*, on the basis of hypothetical mediating effect between physical activity and mathematics through math anxiety. We propose the following hypotheses (see Figure 1):

*Hypothesis 1: Physical activity should be associated with mathematics, and math anxiety partially mediates this association.*

*Hypothesis 2: Physical activity should be associated with mathematics through math anxiety, and perceived parental support and gender could, respectively, play a primary and secondary moderating role in this mediating relationship.*

## Materials and Methods

### Participants and Data Collection

The research data were collected from the national-level education quality assessment of China, which is a national monitoring system for the quality of school education in China, and the subjects tested include mathematics, sciences, Chinese language, physical education, arts, and moral education (Jiang et al., 2019). The data used in this research were specifically drawn from the mathematics component and physical education and health component (PEH) (Wu et al., 2019). Each participant in this study took a mathematics test, a set of physical fitness tests, and two self-reported questionnaires (including mathematics and physical education and health). All questionnaire and exam data for this study were collected on the same day. The sample of this study was distributed across six provinces in eastern, central, and western China. The assessment was conducted simultaneously for every province.

All participants were in the fourth grade (excluding mathematics learning disorders). After the screening and removal of cases with missing values for age, gender, or BMI, 22,509 students (52.9% boys, coded as 0) were included in the present study’s sample. Students were aged 7.42–14.33 years (10.32 ± 0.52), with their schools located in cities (5,598, 24.9%), towns (4,421, 19.6%), and rural areas (12,490, 55.5%).

Fourth-year students were selected as subjects for the following reasons. Firstly, research showed that the association between math anxiety and math achievement starts in childhood – from grades 3 through 5 (Hill et al., 2016), while some other evidence suggested that this association is not significant for 6 to 9-year-olds (Krinzinger et al., 2009), which deserves further attention. Second, the fourth grade was considered a critical period for exploring learning disabilities, children’s learning and habit development. Additionally, since they are more cognitively developed than their younger counterparts, their reading comprehension and written expression are more likely to be reliable (Wu et al., 2019). Third, from the perspective of social support theory, the fourth-grade group with an average age of about 10 is more in line with the research characteristics of parental support (Bandura, 1999; Trost et al., 2003; Gustafson and Rhodes, 2006).

### Measures

#### Mathematics Achievement

The math achievement test came from the national education quality assessment of China. This was developed by mathematicians, mathematics educators, mathematics teaching and research staff, and experienced mathematics teachers from western, central, and eastern China. All the items for the national examination had undergone two pilot tests, multiple rounds (at least three rounds) of expert review, and pre-examination. This applies to the test and all questionnaires below (in this study refers to the mathematics component and physical education and health component).

The fundamental framework contains the following three modules: (a) numbers and algebra, (b) space and shape, and (c) statistics and uncertainty (Ministry of Education of the People’s Republic of China [MOE], 2012). Six parallel tests were used; each of the test booklets was composed of 15 multiple-choice items and 5 construct response items. The internal consistency of the test booklets was above 0.84. Due to the test’s time restriction and the equal weight given to all these modules in the test, a balanced incomplete block design was adopted when the test paper was designed. Each student was randomly assigned to complete one of the six test manuals.

The mathematics tests were assessed using the paper-and-pencil tests. The Rasch model and concurrent calibration were used to link scores of different test booklets to an identical scale provided by Conquest 1.1 (Wu et al., 1998). The item difficulty ranged from −2.270 to 2.597 logits. The total mathematics scores were uniformly converted to an IRT (item response theory) scale, with an average score of 500 and a standard deviation of 100 (Jiang et al., 2019; Wang et al., 2019).

#### Math Anxiety

Math anxiety was measured using the five-item self-reported questionnaire from the mathematic component of the national education quality assessment in China. The collection of questionnaire data was conducted on the same day as the mathematics and physical education tests. These items focused on math anxiety, such as “I am worried that math class would be difficult” and “I am nervous when I have to do math homework”. This instrument employs a 4-point Likert-type scale ranging from 1 (*strongly disagree*) to 4 (*strongly agree*). The responses indicated the extent of the participant’s agreement with each item, with a high score indicating a high level of math anxiety. The mean score of the items ranging from 1 to 4 was used and standardized during analysis. Internal consistency Cronbach’s alpha index was 0.847.

#### Physical Activity Index

The physical activity index was collected from a self-reported questionnaire in the physical education and health component of the national education quality assessment of China on the same day. One of the questions that participants were required to answer related to the frequency of their participation in out-of-school physical activities each week (abbreviated as PAOS in Tables and Figures). The options ranged from 1 to 8, meaning the frequency of student’s participation in out-of-school physical activities per week ranged from 0 to 7 and above. To demonstrate the question’s validity, we selected responses from other closely related questions from the questionnaire of the physical education and health component and identified a series of correlations. The correlation results detailed in Supplementary Appendix 1 indicated that the frequency of physical activity was significantly correlated with several related conditions at a moderate level, especially sweat frequency (*r* = 0.370, *p* < 0.01), physical activity for weekend (*r* = 0.383, *p* < 0.01), intramural physical activity (*r* = 0.330, *p* < 0.01), and duration (*r* = 0.422, *p* < 0.01).

#### Parental Support for Physical Activity

The perceived parental support variable was also adopted from the physical education and health component of the national education quality assessment of China in the questionnaire. The question “Do your parents support your participation in physical activities in your spare time?” was evaluated with response options of “very unsupportive,” “moderately unsupportive,” “moderately supportive,” and “very supportive” (coded as 1–4, respectively). Same to the physical activity index, we selected responses from other closely related questions in the same questionnaire and identified a series of correlations to strengthen the validity of this question. The correlation results detailed in Supplementary Appendix 2 indicate that perceived parental support was significantly correlated with several related conditions at a weak or moderate level.

#### Confounding Variables

In the process of determining the moderated moderated-mediating relationship, SES, school location, and BMI were controlled in the analysis to control for confounding factors in the relation between the implementation of the physical activity index and mathematics through math anxiety (Ericsson and Karlsson, 2014; Hansen et al., 2014; Torrijos-Niño et al., 2014; Alordiah et al., 2015; Li S. et al., 2020).

The association between various socioeconomic variables and academic achievement has been well established (White, 1982; Sirin, 2005; Duncan et al., 2011; Li S. et al., 2020). To determine the participants’ SES, principal component analysis was conducted based on three indicators, namely the highest occupational status of their parents, highest educational level of their parents, and type and amount of possessions in the home (Jiang et al., 2019). The school’s location was used to indicate which part of China (east/central/west) and what type of environment (city/town/rural) the student was living in. The children’s BMI was calculated as BMI = weight (kg)/height^2^ (m^2^) (Fredriks et al., 2000). The age factor was not controlled in this model for two main reasons. First, the entrance age in China is strictly unified by the Ministry of Education; generally, the difference between students in the same academic year is less than 1 year. Second, the proportion of extreme age data was very small (less than 0.6%), and all the key results after adding the age variable were consistent. In accordance with the principle of model simplicity, we did not control for age in this study.

### Statistical Analysis

The descriptive statistics and correlations were calculated using SPSS v19.0 for Windows, and all further analyses were conducted using PROCESS v3.4 for SPSS^1^. In this moderated moderated-mediation model, standardized weekly frequency of out-of-school physical activity and standardized mathematics were the independent variable (*X*) and dependent variable (*Y*), respectively; standardized math anxiety was an intermediary variable (M); and standardized perceived parental support for physical activity and gender were the primary and secondary moderators (*W* and *Z*), respectively. School location, SES, and BMI were the control variables. The core continuous variables were standardized during data analysis, including math achievement, math anxiety, weekly frequency of out-of-school physical activity and parental support. The regression coefficients presented were standardized regression coefficients in the mediation model results, and were unstandardized regression coefficients in the moderated moderated-mediation model results (PROCESS software can only provide standardized regression coefficients for mediation-only models).

The statistical model of the moderated moderated-mediation model can be represented by the following two equations (Hayes, 2018; Hayes and Rockwood, 2020):


$$\hat{M}=i_{M}+a_{1}\,X+a_{2}\,W+a_{3}\,Z+a_{4}\,X\,W$$

(1) $$+a_{5}\,X\,Z+a_{6}\,W\,Z+a_{7}\,X\,W\,Z$$

(2) $$\hat{Y}=i_{Y}+c^{\prime }\,X+b\,M$$

**FIGURE 2:**
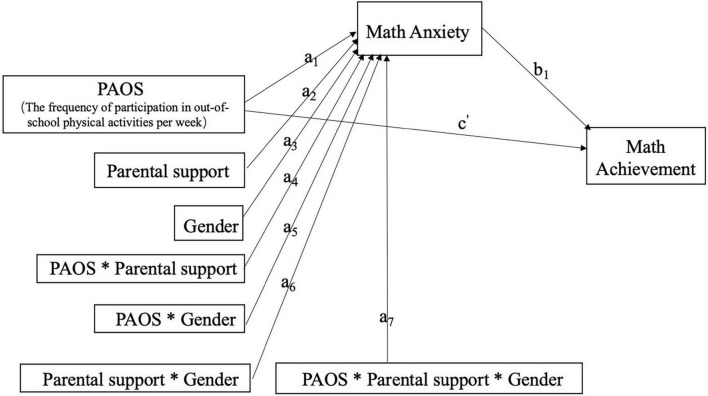
Statistical model of the moderated moderated-mediation model.


The coefficients in the model are shown in Figure 2. From Eq. (1), the effect of X on M can be expressed as:

(3) $$\theta_{X\to M}=a_{1}+a_{4}\,W+a_{5}\,Z+a_{7}\,W\,Z$$

Multiplication of the effect of X on M and the effect of M on Y yields the indirect effect of X on Y through M:


$$\theta_{X\to M}\,b=\left(a_{1}+a_{4}\,W+a_{5}\,Z+a_{7}\,W\,Z\right)\,b$$

(4) $$=a_{1}\,b+a_{4}\,b\,W+a_{5}\,b\,Z+a_{7}\,b\,W\,Z$$

Through algebraic manipulation, Eq. (4) can be written in the following equivalent form:


(5) $$\theta_{X\to M}\,b=a_{1}\,b+\left(a_{4}\,b+a_{7}\,b\,Z\right)\,W+a_{5}\,b\,Z$$

The slope of the line relating W to the indirect effect of X is a_4_b + a_7_bZ, which is called the index of conditional moderated mediation by W in this model. The coefficient values had a one-to-one correspondence in the results.

## Results

### Descriptive Statistics and Correlations

The descriptive statistics are listed in Table 1, which includes the mean and standard deviations of the core variables (school location, SES, gender, age, BMI, mathematics, math anxiety, parental support, and weekly frequency of out-of-school physical activity) are presented. As described in Table 1, mathematics had significant moderate or weak negative correlations with math anxiety (*r* = −0.376, *p* < 0.01) and school location (*r* = −0.239, *p* < 0.001) and significant moderate positive correlations with SES (*r* = 0.418, *p* < 0.01). Math anxiety exhibited a significant weak correlation with weekly frequency of out-of-school PA (*r* = −0.121, *p* < 0.001) and parental support (*r* = −0.172, *p* < 0.01). Parental support was significantly correlated with all core variables; it exhibited weak correlations with weekly frequency of out-of-school PA (*r* = 0.228, *p* < 0.01), math anxiety (*r* = −0.121, *p* < 0.01), and SES (*r* = 0.113, *p* < 0.01).

**TABLE 1:** Descriptive statistics and correlations of core variables.

	Min	Max	Mean	*SD*	1	2	3	4	5	6	7	8
(1) Mathematics	126.72	834.83	513.15	125.80	1.000							
(2) Math anxiety	1.00	4.00	2.02	0.82	−0.376**	1.000						
(3) SES	–3.08	3.29	0.00	1.05	0.418**	−0.232**	1.000					
(4) School location	1.00	3.00	2.31	0.84	−0.239**	0.152**	−0.453**	1.000				
(5) Gender	0.00	1.00	0.47	0.50	−0.048**	0.054**	–0.008	0.016*	1.000			
(6) BMI	8.30	50.30	17.14	3.06	0.069**	−0.044**	0.133**	−0.106**	−0.129**	1.000		
(7) PAOS	1.00	8.00	4.51	2.26	0.138**	−0.172**	0.165**	−0.082**	−0.023**	0.029**	1.000	
(8) Parental support	1.00	4.00	3.37	0.83	0.082**	−0.121**	0.113**	−0.062**	0.052**	0.033**	0.228**	1.000

Parental support, perceived parental support for their children’s participation in out-of-school sports activities; PAOS, the frequency of participation in out-of-school physical activities per week. These variables were standardized during data analysis: math achievement, math anxiety, physical activity, parental support. *p < 0.05, **p < 0.01, ***p < 0.001.

### Mediating Effect of Math Anxiety

Table 2 presents the direct, indirect, and total effects of mathematics anxiety as a mediator in the relationship between physical activity and mathematics performance in great detail. According to the standardized regression results, the effect of physical activity frequency per week on math anxiety was significant (β = 0.136, *p* < 0.001; LLCI = −0.153; ULCI = −0.131), and the effect of math anxiety on mathematics was also significant (β = 0.278, *p* < 0.001; LLCI = −45.748; ULCI = −42.163). According to the results of mediating effects, a significant indirect effect of weekly frequency of out-of-school physical activity on mathematics through math anxiety (Indirect effect: *m* = *ab* = 2.117, *p* < 0.001; LLCI = 1.945; ULCI = 2.404; *R*_*M*_ = 56.62%) was observed; a 95% C.I. was obtained with 5,000 bootstrapping resamples. The direct effect of weekly frequency on mathematics was significant [Direct effect: *c*′ = 1.668(0.327), *p* < 0.001; LLCI = 1.026; ULCI = 2.309; *R*_*M*_ = 43.38%], indicating a partial mediation relationship between weekly frequency and mathematics through math anxiety. In the total effect model [*c* = *c′* + *ab* = 3.845(0.340), *p* < 0.001; LLCI = 3.178; ULCI = 4.512], weekly frequency of out-of-school physical activity exhibited a significant positive relation to mathematics. The signs of *ab* and *c*′ were the same, and the effect size index *p*_*m*_ = *ab*/*c* (range from 0 to 1) indicated the proportion of the mediation effect to the total effect (Preacher and Kelley, 2011; Wen and Fan, 2015). The effect size index *p_m* in this mediation model was 0.566, meaning that the mediating effect of math anxiety accounts for 56.6% of the total effect between physical activity and mathematics.

**TABLE 2:** Mediation analysis of the association between PAOS and math achievement through math anxiety.

	Math anxiety				Math achievement (total effect model)				Math achievement (direct effect model)			
			
Variables	β (*S.E*.)	*P*	95% C.I.	*R* ^2^	β (*S.E.*)	*P*	95% C.I.	*R* ^2^	β (*S.E*.)	*P*	95% C.I.	*R* ^2^
PAOS	**−0.136**	0.000***	**−0.153**	0.078***	**0.069**	0.000***	**3.178**	0.186***	**0**.**030**	0.000***	**1.026**	0.261***
	**(0.002)**		**−0.131**				**4.512**		**(0.327)**		**2.309**	
SES	−0.181	0.000***	–0.153		0.375	0.000***	43.524		0.324	0.000***	37.339	
	(0.006)		–0.131				46.759		(0.796)		40.462	
School_location1	0.003	0.721	–0.026		0.011	0.117	–0.907		0.012	0.078	–0.440	
	(0.016)		0.037				8.147		(2.200)		8.183	
School_location2	0.064	0.000***	0.077		–0.066	0.000***	–20.613		−0.048	0.000***	–15.823	
	(0.014)		0.133				–12.629		(1.942)		–8.210	
BMI	−0.003	0.646	–0.004		0.004	0502	–0.324		0.003	0.577	–0.336	
	(0.002)		0.003				0.662		(0.240)		0.604	
Gender	0.048	0.000***	0.057		–0.041	0.000***	–13.362		-0.028	0.000***	–9.787	
	(0.011)		0.099				–7.366		(1.458)		–4.071	
Math anxiety	–	–	–		–	–	–		**−0.287**	0.000***	**−45.748**	
									**(0.915)**		**−42.163**	

*p < 0.05, **p < 0.01, ***p < 0.001.

*PAOS, the frequency of participation in out-of-school physical activities per week; school location 1, comparison of school location between town and city; school location 2, comparison of school location between rural area and city.*

*These variables were standardized during data analysis: Math achievement, Math anxiety, Physical activity, Parental support.*

*The standardized regression coefficients β are presented in this table.*

*The bolded coefficient values are the key results in this mediation model.*

Consequently, the mediating effect of weekly frequency of out-of-school physical activity on mathematics through math anxiety was statistically significant, and Hypothesis 1 was fully supported (see Table 2). The effect size index *f*^2^ = *R*^2^/(1−*R*^2^) was used to measure the power of the mediation model, *f*^2^ values equal to 0.02, 0.15, and 0.35 correspond to small, medium, and large effect sizes, respectively (Cohen, 2009). The effect size of the mediation model to math anxiety $f_{t\,o\,M\,A}^{2}=0.078/\text{(1-0.078)}=0.085$ was moderately small. The direct influence to math achievement of the mediation model was with a large effect size $f_{D\,i\,r\,e\,c\,t-M\,A\,T\,H}^{2}=0.261/\left(1-0.261\right)=0.353$. The total influence to math achievement of the mediation model was with a medium effect size $f_{T\,o\,t\,a\,l-M\,A\,T\,H}^{2}=0.186/\left(1-0.186\right)=0.229$.

### Moderated Moderated-Mediation Model

Hypothesis 2 pertained to the conditional process model, which featured moderated moderated-mediation effects. The effect of parental support for physical activity and the child’s gender on weekly frequency of out-of-school physical activity and mathematics through math anxiety were tested simultaneously (Hayes, 2018). The unstandardized results revealed that neither the three-way interactions of weekly frequency × parental support × gender nor the two-way interactions of weekly frequency × gender were significant, whereas the interactions of weekly frequency × parental support and parental support × gender were significant. The effect of parental support and children’s gender on mathematics through math anxiety (*b* = −36.239, *p* < 0.001) accounted for 29% of the variance (*F*[11,22497] = 187.202, *p* < 0.001; Table 3). In addition, weekly frequency of out-of-school physical activity had a positive significant effect on mathematics and a negative effect on math anxiety (Table 3).

**TABLE 3:** Moderated moderated-mediation model results and regression results for math anxiety as an intermediary variable.

		Outcome						
		
		M: Math anxiety				Y: Math achievement		
				
Predictor		*B*	*SE*	*P*		*B*	*SE*	*P*
PAOS	a_1_ →	–0.116	0.009	0.000***	*c*′→	3.808	0.738	0.000***
Parental support	a_2_ →	–0.064	0.009	0.000***				
Gender	a_3_ →	0.103	0.013	0.000***				
PAOS × PS	a_4_ →	–0.032	0.008	0.000***				
PAOS × Gender	a_5_ →	–0.008	0.013	0.553				
PS × Gender	a_6_ →	–0.035	0.014	0.010*				
PAOS × PS × Gender	a_7_ →	0.004	0.013	0.735				
SES		–0.167	0.007	0.000***		38.800	0.797	0.000***
BMI		–0.001	0.002	0.772		0.280	0.238	0.239
Town-city		0.003	0.020	0.893		3.962	2.201	0.072
Rural area-city		0.125	0.017	0.000***		–12.039	1.943	0.000***
Math anxiety					*b*→	–36.239	0.750	0.000***
	*R*	0.290		0.000***		0.511		0.000***

**Gender**	***Z*-score-PS**	**Conditional effect**	***SE***				**LICI**	**ULCI**

Female/daughter	-1	3.053	0.448				2.202	3.969
Female/daughter	0	4.211	0.342				3.553	4.888
Female/daughter	1	5.096	0.426				4.264	5.958
Male/son	-1	3.497	0.564				2.407	4.613
Male/son	0	4.495	0.380				3.761	5.255
Male/son	1	5.259	0.469				4.363	6.209

*p < 0.05, **p < 0.01, ***p < 0.001.

*PAOS, the frequency of participation in out-of-school physical activities per week; parental support, perceived parental support for their children’s participation in out-of-school physical activities.*

*These variables were standardized during data analysis: math achievement, math anxiety, physical activity, parental support.*

*The unstandardized coefficients B are presented in this table (PROCESS software can only provide standardized regression coefficients for mediation-only models).*

Specifically, the index of the whole moderated moderated-mediation model did not reach the significance level [Index = −0.159 (0.500); LLCI = −1.135; ULCI = 0.820]; however, the indices of the conditional moderated mediation with the parental support variable were significant for both male and female children [The conditional index of male = 1.158 (0.309); LLCI = 0.544; ULCI = 1.763; the conditional index of female = 0.999 (0.393); LLCI = 0.230; ULCI = 1.780]. This indicated that math anxiety played a significant mediating role at different levels of perceived parental support for both daughters and sons.

**FIGURE 3:**
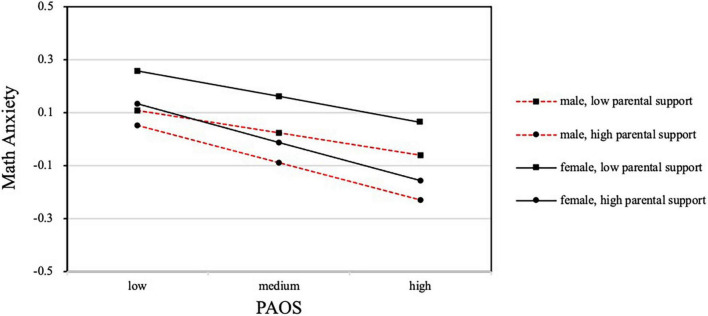
Math anxiety as a function of PAOS, parental support, and children’s gender.

Figure 3 illustrates the effect of weekly frequency of out-of-school physical activity on mathematics through math anxiety, which was significant for both boys and girls in terms of parental support. From the overall trend, the anxiety level decreased significantly with the increase of weekly frequency of out-of-school physical activity, and most girls had a higher anxiety level than boys with the same level of parental support. The interaction between boys with low parental support and girls with high parental support was significant. In other words, in the same low weekly frequency situation, girls had higher levels of anxiety than boys; however, with the increase in the frequency of weekly out-of-school physical activity, the anxiety level of girls with high parental support was significantly lower than that of boys with low parental support.

**FIGURE 4:**
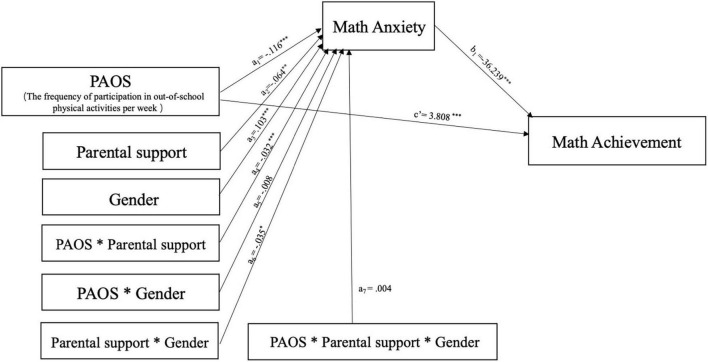
Statistical (path diagram) form of the fourth-grade participants. *p < 0.05, **p < 0.01, ***p < 0.001.


Additionally, Figure 4 depicts a statistical path diagram for the fourth-grade participants. According to the coefficients B, Eq. (5) can be written in the following equivalent form:

$$\theta_{P\,A\,O\,S\to a\,n\,x\,i\,e\,t\,y}\,b=4.204+\left(1.160-0.145\,g\,e\,n\,d\,e\,r\right)\,p\,a\,r\,e\,n\,t\,a\,l\,s\,u\,p\,p\,o\,r\,t+0.290\,g\,e\,n\,d\,e\,r.$$

$$\theta_{P\,A\,O\,S\to a\,n\,x\,i\,e\,t\,y}\,b$$

=

4.204+(1.160-0.145⁢g⁢e⁢n⁢d⁢e⁢r)

p⁢a⁢r⁢e⁢n⁢t⁢a⁢l⁢s⁢u⁢p⁢p⁢o⁢r⁢t+0.290⁢g⁢e⁢n⁢d⁢e⁢r.

**FIGURE 5:**
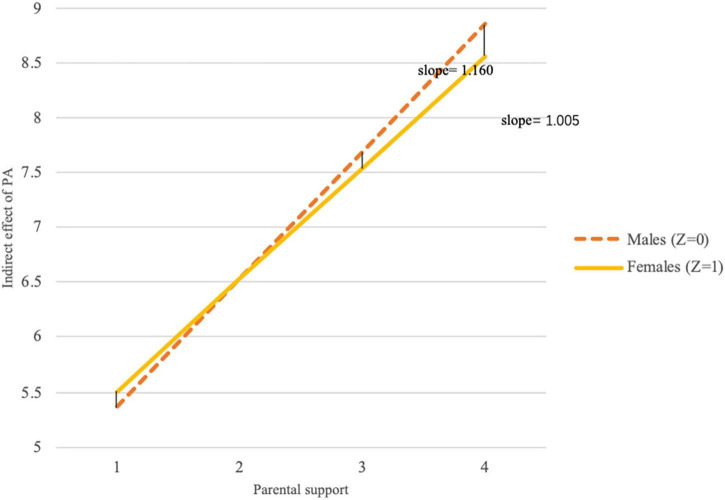
Visual depiction of the indirect effect of PA on mathematics through math anxiety as a function of parental support (W) and gender (Z).

The results showed that the mediating effect increased positively with *W* (perceived parental support), regardless of whether *Z* (gender) was 0 (male) or 1 (female). These results are visually reflected in Figure 5. The effect size of the moderated moderated-mediation model to math anxiety $f_{\text{M-M}-M-M\,A}^{2}=0.290^{2}/\left(1-0.290^{2}\right)=0.082$ was moderately small, while the whole moderated moderated-mediation model had a large effect size to math achievement, $f_{\text{M-M}-M-M\,A\,T\,H}^{2}=0.511^{2}/\left(1-0.511^{2}\right)=0.353$.

## Discussion

In this study, a moderated moderated-mediation model was used to explore the association between physical activity and academic performance. Its contributions are three-fold. First, our study verified that out-of-school physical activity can affect mathematics. Second, we found that physical activity influenced mathematics through the mediating role of math anxiety. Finally, we observed that the effect of out-of-school physical activity on math anxiety was contingent on the perceived parental support for their participation in physical activity, regardless of the child’s gender.

### Out-of-School Physical Activity and Academic Performance

Consistent with our initial hypothesis, the results from our analysis of data from a representative sample of Chinese students supported the significant and direct effect of out-of-school physical activity on mathematics and were similar to other studies on such intramural activities (Donnelly et al., 2009; Diamond, 2015). Our results are also consistent with those of other studies that have demonstrated that in-school physical activity positively affects mathematical skills (Fredericks Claude et al., 2006; Davis et al., 2011; Erwin et al., 2012). The effect size of direct influence to math achievement of the mediation model meant that our research provided evidence for the positive impact of physical activity on academic performance from the perspective of physical activity out-of-school.

The positive effect of physical activity on academic performance is supported by the explanation of physiological performance and further verified the executive function hypothesis (Kopp, 2012). Regular exercise and moderate aerobic activity are associated with greater brain volume, improved neurophysiological responses to stimulation, and better levels of growth factors that promote brain tissue growth, neurogenesis, and angiogenesis, with physiological indicators like the flow of blood and oxygen to their brain concerning the effects of exercise on cognitive function (Teferi, 2020). Research confirmed that regular physical activity alters specific brain structures and functions, especially in tests that require more executive function (Miyake et al., 2000; Diamond, 2013), which is a subset of goal-directed cognitive operations underlying perception, memory, and action (Donnelly et al., 2016). Academic performance is strongly associated with neurocognitive improvements, and are closely related to core executive functions (cognitive flexibility, working memory, and inhibition) during brain development (Diamond, 2012; Singh et al., 2012; Álvarez-Bueno et al., 2017). The positive effect of physical activity on executive function has also been demonstrated (Diamond, 2012; van der Niet et al., 2014; Ishihara et al., 2017; de Bruijn et al., 2018). Hence, physical activity may prove to be a simple, yet important, method of enhancing those aspects of children’s intelligence, cognition, and academic achievement (Tomporowski et al., 2007). The results of this study validated that out-of-school physical activity is equally effective.

The present study’s focus on out-of-school physical activity expands the literature in this area and highlights the role of extramural sports. The impact of physical activity on academic performance and cognitive development is often underestimated, and out-of-school sports are given especially little attention. This is possibly because when researchers initially explored the impact of physical activity from the perspective of academic performance, they have tended to pay more attention to school education and focus on intra-school physical education, ignoring the positive impact of out-of-school physical activity on academic achievement. Compared with physical education in school, out-of-school physical activity is more in line with students’ interests, the sports environment and types are more diverse, the exercise time is relatively free, the atmosphere is more relaxed, and its psychological and physical effects cannot be ignored. Combining the previous results on physical activity in schools and the factor of family support, this study informs how physical activity can have a more comprehensive effect on children’s mental health, physical health, and academic performance.

Nonetheless, there is still room for improvement regarding the research design of physical activity indicators. On the one hand, the findings of this study were based on large-scale data, and some indicators (physical activity, and parental support for physical activity) were derived from fewer items. The study’s replicability and the extension of this conclusion need to be further verified in other studies. On the other hand, almost all studies focus on one aspect of intramural or extramural sports, possibly due to the limitations of research design or practical operation (Donnelly et al., 2016; Álvarez-Bueno et al., 2017; Barbosa et al., 2020). When studying individual differences, in addition to the regular intramural sports curriculum, other intramural sports should be considered, which can make the role of sports in the research more refined.

Further, it is worth emphasizing that the impact of physical activity on other kinds of academic performance is also of concern. Although the subject of mathematics has received the most attention (Barbosa et al., 2020), there are several studies on reading, spelling, language, science, and geography. Most of these studies have also focused on intramural physical education (Sibley and Etnier, 2003; Esteban-Cornejo et al., 2015; de Greeff et al., 2018; Bedard et al., 2019). From the meta-analyses, physical activity had null or small-to-medium positive effects on academic performance, and chronic physical activity showed a moderately positive effect on academic performance, but acute physical activity did not demonstrate benefits (Barbosa et al., 2020). This deserves further attention, whether it is for different disciplines or different forms of physical activity.

### Math Anxiety and Its Role in Physical Activity and Academic Performance

Our research provided further evidence for the distraction hypothesis, in which math anxiety can be regarded as similar to other mental health factors, for example, self-concept (Dapp and Roebers, 2019) and self-efficacy (Suchert et al., 2016; Liu et al., 2020), that improve the relationship between physical activity and academic performance as a mediator with the effect size of the proportion of the mediation effect to the total effect, which means that physical activity outside of school can improve math performance to some extent by relieving math anxiety, but not as strongly.

The present study’s findings on the positive improvement effect of physical activity on anxiety levels provide further reference and highlight the characteristics of out-of-school physical activity. Systematic meta-analyses from the 1990s previously established a significant and negative correlation between mathematics anxiety and mathematics achievement, although some meta-analyses have recorded inconsistent effects of physical activity on anxiety (Craft and Landers, 1998; Ahn and Fedewa, 2011; Cerrillo-Urbina et al., 2015; Ferreira-Vorkapic et al., 2015). This study’s analysis of the effect of out-of-school physical activity is more in line with the impacts on physical and mental health. Off-campus sports activities usually initiate from students’ interests and are characterized by more freedom in exercise intensity and duration. It is, thus, conceivable that they could have a positive psychological and physiological effect.

The findings with a medium effect size provide evidence for the conceptual model, which hypothesized that the mechanisms explaining the association between physical activity, cognition and mental health in young people might be neurobiological, psychosocial and/or behavioral, and might be affected by the indicators of physical activity (Lubans et al., 2016; Barth Vedøy et al., 2020). The effects of physical activity on mental health can be explained by physiological indicators, such as cerebral blood flow and arousal levels (Querido and Sheel, 2007), neurotransmitters (Ploughman, 2008), the growth and plasticity of neurons (Hassevoort et al., 2016), and measures of brain function related to executive function (Donnelly and Lambourne, 2011). Results were similar to another study, it showed that increased physical activity levels and fitness can help alleviate or relieve depression, anxiety and stress by improving bone and musculoskeletal function (Eveland-Sayers et al., 2009). And the findings from previous large-scale observational studies also suggest that physical activity participation has a small to moderate effect on preventing and managing the risk of anxiety and depression, which in turn affects academic performance and mental health (Teferi, 2020).

Although the effect of physical activity on anxiety was supported in this study, other mental health factors were not considered or controlled for, which may be a reason behind the weak effect on this aspect. In particular, general and test anxiety, which are closely related to math anxiety, (Hunsley, 1987), were not considered. Since general anxiety is not specific to a situation, its relationship to mathematics performance is less direct than math anxiety (Barlow et al., 1986). Regarding test anxiety, since this assessment was a low-stakes test, the purpose was to monitor the overall levels, and not fed the results back to students. Studies on the relationship between general anxiety and test anxiety with mathematics avoidance behavior showed that, compared with situational measures of test anxiety, mathematics anxiety was associated with higher ability and mathematics avoidance behavior (Dew et al., 1984). Therefore, general anxiety and test anxiety were not controlled in this study. However, the influence of these two on performance cannot be ignored, and it is necessary to obtain supplementary verification in follow-up research.

In addition, evidence suggests that the association between math anxiety and math achievement starts in childhood, and remains significant through adulthood (Hill et al., 2016). As for adolescents and young adults, a large body of research has found small-to-moderate negative correlations between math anxiety and math achievement in middle school, high school, and undergraduate student samples (Hembree, 1990; Ma, 1999). The difference in the magnitude of this relationship at different growth stages needs to be further explored. Moreover, Lagerberg (2005) argued that physical activity cannot guarantee an improvement in mental health because many moderating factors are present. The factors that moderate the relationship between students’ math anxiety and achievement include gender, school grade, age, ethnicity, teachers’ characteristics, and (low) math ability (Lagerberg, 2005; Barroso et al., 2021). These influencing factors deserve further refinement or combination in subsequent studies.

### Moderating Role of Parental Support According to Children’s Gender

Notably, parental support for children’s out-of-school physical activity had a significant positive effect on reducing anxiety and improving mathematical performance for both boys and girls. The results regarding the interaction between perceived parental support and gender indicated that although the average math anxiety levels among girls with low weekly frequency of out-of-school physical activity were higher than that of boys, as children perceive more support for physical activity from their parents, the anxiety levels of girls with high parental support were significantly lower than those of boys with low parental support. Thus, parents’ support for their children’s after-school physical activity played a key, positive role in improving their children’s mental health and academic performance. Naturally, it will also contribute to their physical health, which deserves more attention from parents and requires schools and relevant education departments to provide corresponding coordination and assistance.

The interaction between gender and weekly frequency of out-of-school physical activity had no significant effect on anxiety, indicating that out-of-school physical activity can improve math anxiety and mathematical performance regardless of gender. Although Hypothesis 2 was not verified, we obtained research results that can be beneficial to improving children’s anxiety and academic performance. This means that if parents give support for either their children’s physical activity, children can alleviate their anxiety through appropriate physical activities and improve mathematics performance. Therefore, parents should be encouraged to support the moderate-to-vigorous physical activity of their children, and different methods can be considered according to children’s gender (Lijuan et al., 2017).

In addition, the significant interaction between parental support and weekly frequency of out-of-school physical activity on anxiety indicated that different levels of parental support for their children’s extracurricular physical activity significantly affected children’s math anxiety and mathematics. The results showed that the whole moderated moderated-mediation model had a large effect size to math achievement. It can be suggested that within a reasonable range (e.g., once a day), the higher the degree of parental support for their physical activity that children perceive, the more extensive the effect of physical activity on relieving their anxiety, which in turn affects their academic performance. It is recommended that education practitioners and parents can focus on supporting their children’s physical activity in different aspects.

### Theoretical and Practical Implications

This study has implications for theory and educational practice and application and provides empirical evidence for the positive impact of physical activity on mental health and subject education. First, our research provided further support for the distraction hypothesis, the executive function hypothesis and verified the mediation effect of anxiety. Appropriate levels of physical activity had a significant effect on improving academic performance through the alleviation of anxiety, which supported our initial hypothesis and was consistent with the mediating effect of other psychological variables in relevant previous studies. The significant partial mediation effect of math anxiety further validated this mechanism.

Second, our results provided evidence for the positive effect of physical activity on mathematics academic performance from the specific perspective of after-school physical activity, which has important reference value for enhancing the prioritization of physical activity by education departments. It is not so easy to attract attention to the importance of the effect of out-of-school physical activity, especially among those parents who neglect physical activity or overemphasize academic performance; however, it is worthwhile to help them realize that appropriate physical activity is a good way to benefit both their children’s academic performance and physical health. Parents should be made aware of the potential harm of physical and mental problems. Rather than focusing solely on the academic performance of their children or condoning their children to spend too much time on electronic products, parents should try to make changes in their thinking and daily arrangements and support their children as much as possible to participate in physical activities.

Third, the nexus between physical activity and mathematics through math anxiety has crucial implications for policymakers. Governments should emphasize the indispensable role of physical activity, assist schools in formulating feasible physical education and activity plans, and provide corresponding financial and implementational support to reduce the negative effects of overlearning at the national level. Educational departments at different levels can support physical activity in schools according to the actual situation and reasonably increase courses and extracurricular activities in schools, including curricular physical education (PE), integrated physical activity (active breaks or teaching subjects such as math with physically active tasks), and extracurricular physical activity (active recess or lunchtime physical activity).

Fourth, the communication between schools and families should be enhanced, with schools advocating for the value of physical activity to parents. Schools should be encouraged to confirm the positive role of physical activity to parents. While actively organizing and arranging intra-school physical activity, parents should be able to actively and reasonably participate in children’s physical activity arrangements. This would allow the effective combination of physical activity both in and out of school, rendering the impact of physical activity on physical and mental health more efficient.

Lastly, to encourage physical activity among children, the environment for physical activities must be engaging, rather than based on strict discipline and an emphasis on skill, otherwise, it may cause children further psychological burden (Lagerberg, 2005). Moreover, while encouraging physical activity, parents should be cautious of over-emphasizing its importance, which would lead to replacing one misunderstanding with another. The special needs, interests, and mental health of children should be taken into consideration; extra training courses should be selected carefully; and compulsory activities must be avoided. Furthermore, policymakers and education executors should strengthen their participation in, and guidance they provide on, the environment, content, and implementation of physical activity in order to obtain more reliable evidence and improve the impact of physical activity on children’s physical health, psychological health, and academic performance more effectively.

### Limitations

Some limitations of the current study should be mentioned. First, part of the analyzed data was derived from self-reported questionnaires. For children in the fourth grade, it is inevitable that this data would include inaccurate or invalid answers. In follow-up research, more flexible and objective data collection methods should be adopted, such as online check-ins or activity records. Second, the variety of key variables analyzed could be richer. Our study used the frequency of activity as a variable when assessing out-of-school physical activity; however, the type of activity (e.g., aerobic), duration, and dosage can be investigated by future studies on the benefits of physical activity (Petruzzello et al., 1991; Ahn and Fedewa, 2011; Kyan et al., 2019). In addition, in terms of parental support variables, further angles of inquiry could include the supportive behavior of parents, degree of parental participation, and maternal or paternal logistical support (Trost et al., 2003; Davison, 2004; Gustafson and Rhodes, 2006). Third, more diverse experimental designs and a richer composition of participants can be considered in order to compare and supplement the present study’s research results. The intervention effects of physical activity on mental health may differ depending on other moderators such as research design (Conn, 2010) and study implementation (Stice et al., 2006), in addition to gender (Kremers et al., 2007; Simen-Kapeu and Veugelers, 2010). In future research, more moderating variables or the comparative study of different groups can be used. Controlling conditions should be more stringent as well (e.g., control for physical activity in other contexts, general anxiety, or test anxiety) to undertake richer and more systematic research on the relationships between physical activity, psychological variables, and other academic achievements.

## Conclusion

This research examined whether physical activity improves math achievement through the alleviation of math anxiety, with the incorporation of the moderating role of perceived parental support for physical activity and the factor of gender. The results demonstrated that both boys and girls can benefit significantly from parentally supported physical activity, which can alleviate math anxiety and, in turn, improve their academic performance in mathematics. As perceived parental support for physical activity and the frequency of participation in out-of-school physical activities per week increased, the children’s anxiety levels and mathematics were significantly affected in a positive direction.

## Data Availability Statement

The data analyzed in this study is subject to the following licenses/restrictions: the dataset is confidential. Further inquiries should be directed to the corresponding author.

## Ethics Statement

The studies involving human participants were reviewed and approved by Collaborative Innovation Center of Assessment Toward Basic Education Quality, Beijing Normal University, Beijing, China. Written informed consent from the participants’ legal guardian/next of kin was not required to participate in this study in accordance with the national legislation and the institutional requirements.

## Author Contributions

TY supervised the quality of the research process and provided financial support. HL supervised the quality of the research process and revised it critically for important intellectual content. JZ designed the study, analyzed the data, and wrote the manuscript. HW provided valuable suggestions for the study design and checked the quality of the research. XW participated in the data analysis and manuscript revision. YW participated in the data analysis and provided suggestions. All authors contributed to the article and approved the submitted version.

## Conflict of Interest

The authors declare that the research was conducted in the absence of any commercial or financial relationships that could be construed as a potential conflict of interest.

## Publisher’s Note

All claims expressed in this article are solely those of the authors and do not necessarily represent those of their affiliated organizations, or those of the publisher, the editors and the reviewers. Any product that may be evaluated in this article, or claim that may be made by its manufacturer, is not guaranteed or endorsed by the publisher.

## Footnotes

1
http://processmacro.org/index.html
