# Supplementary material for: The Association Between Physical Activity and Mathematical Achievement Among Chinese Fourth Graders: A Moderated Moderated-Mediation Model
Source: Front Psychol. 2022 May 9;13:862666. doi: 10.3389/fpsyg.2022.862666 (PMC9126125; doi:10.3389/fpsyg.2022.862666)
Supplement: Supplementary file 1 [file Data_Sheet_1.pdf]

The Association Between Physical Activity and Mathematical Achievement Among Chinese Fourth Graders: A Moderated Moderated-Mediation Model

**Appendix Captions**

Appendix 1. Correlations table of physical activity
Appendix 2. Correlations table of parental support for physical activity

Appendix 1. Correlations table of physical activity

| | 1 | 2 | 3 | 4 | 5 | 6 | 7 |
|---|---|---|---|---|---|---|---|
| 1. PAOS | 1.000 | | | | | | |
| 2. Parental support | 0.228** | 1.000 | | | | | |
| 3. Sweat frequency | 0.370** | 0.111** | 1.000 | | | | |
| 4. Interest classes | 0.255** | 0.180** | 0.163** | 1.000 | | | |
| 5. PA for weekend | 0.383** | 0.211** | 0.241** | 0.344** | 1.000 | | |
| 6. Intramural PA: | 0.330** | 0.226** | 0.210** | 0.446** | 0.340** | 1.000 | |
| 7. Duration | 0.422** | 0.178** | 0.249** | 0.233** | 0.360** | 0.227** | 1.000 |

**p* < .05, ***p* < .01, ****p* < .001

**1. PAOS:** The frequency of participation in out-of-school physical activities per week (0–7 and above); 2. **Parental support:** Perceived parental support for their children's participation in out-of-school physical activities (1–4); 3. **Sweat frequency**: In the past 7 days, except for physical education classes and extracurricular sports activities organized by the school, how many times did you exercise continuously for 30 minutes or more and your body sweated (0–7 and above)? 4. **Interest classes**: The frequency of participation in off-campus sports interest classes per week (0–3 and above); 5. **PA for weekend**: On Saturday and Sunday, how many days do you usually do physical activities (including interest classes and independent physical exercise, etc.) for a total of 60 minutes (0–2)? 6. **Intramural PA:** The frequency of participation in intramural sports activities per week (0–5)； 7. **Duration:** Duration of each physical activity (no exercise = 1, 30 min or less = 2, 30 min to 1 h = 3; more than 1 h = 4).

Appendix 2. Correlations table of parental support for physical activity

| | 1 | 2 | 3 | 4 | 5 | 6 | 7 |
|---|---|---|---|---|---|---|---|
| 1. BMI | 1.000 | | | | | | |
| 2. Parental support | 0.031** | 1.000 | | | | | |
| 3. Parents’ exercise | 0.023** | 0.355** | 1.000 | | | | |
| 4. PAOS | 0.040** | 0.242** | 0.214** | 1.000 | | | |
| 5. Duration | 0.067** | 0.197** | 0.166** | .414** | 1.000 | | |
| 6. Intramural PA | 0.001 | 0.198** | 0.201** | .293** | .209** | 1.000 | |
| 7. Interest classes | 0.001 | 0.193** | 0.215** | .244** | .215** | .383** | 1.000 |

*$p < .05$, **$p < .01$, ***$p < .001$

**1. BMI:** Body mass index; 2. **Parental support:** Perceived parental support for their children’s participation in out-of-school physical activities (1–4). 3. **Parents’ exercise:** Parents’ participation in sports activities in their spare time (neither = 1, father = 2, mother = 3, both = 4); 4. **PAOS:** the frequency of participation in out-of-school physical activities per week (0–7 and above); 5. **Duration:** Duration of each physical activity (no exercise = 1, 30 min or less = 2, 30 min to 1 h = 3; more than 1 h = 4); 6. **Intramural PA:** The frequency of participation in intramural sports activities per week (0–5); 7. **Interest classes:** The frequency of participation in off-campus sports interest classes per week (0–3 and above).
